# Partitioning variation in measurements of beef carcass traits using ultrasound^1^

**DOI:** 10.1093/tas/txaa162

**Published:** 2020-09-01

**Authors:** Bradie M Schmidt, Michael G Gonda, Michael D MacNeil

**Affiliations:** 1 Department of Animal Science, South Dakota State University, Brookings, SD; 2 Department of Animal, Wildlife and Grassland Sciences, University of the Free State, Bloemfontein, South Africa; 3 Delta G, Miles City, MT

**Keywords:** beef, carcass merit, imaging laboratory, ultrasound

## Abstract

Ultrasound technology provides cattle breeders with a quick, noninvasive, and inexpensive way to measure carcass data on live animals. Ultrasound data are used as indicator traits in cattle genetic evaluations for economically relevant carcass traits. Ultrasound cattle genetic evaluations assume homogeneous additive genetic and residual variance. Thus, the objective was to partition phenotypic variance in ultrasound carcass measurements into components for additive genetic effects, technicians, contemporary groups within technicians, and residual and to examine the homogeneity of these variances among image interpretation laboratories. Records of longissimus muscle area (LMA), percentage of intramuscular fat (IMF), and subcutaneous fat depth (SFD), measured using ultrasound, were provided by the American Angus Association (*n* = 65,967), American Hereford Association (*n* = 43,182), and American Simmental Association (*n* = 48,298). The data also included contemporary group, technician, imaging lab, and a three-generation pedigree for each animal. Variance components for ultrasound carcass measurements were first estimated with univariate animal models for each breed and imaging laboratory using derivative-free restricted maximum likelihood. Then, treating data from each imaging laboratory as separate traits, genetic correlations between laboratories for LMA, percentage of IMF, and subcutaneous fat were estimated with trivariate animal models. The technician explained 12–27%, 5–23%, and 4–26% of the variance for IMF, SFD, and LMA, respectively, across all three breeds. Variance due to technician was often greater than variance due to additive genetic effects but almost always less than that explained by the contemporary group. Within breeds, estimates of additive genetic variance for LMA, SFD, and IMF differed (range divided by mean) among laboratories by 4.5%, 21.5%, and 39.4 % (Angus); 31.6%, 15.0%, and 49.1% (Hereford); and 19.9%, 46.6%, and 55.3% (Simmental), respectively. Likewise, estimates of residual variance for LMA, SFD, and IMF differed among laboratories by 43.4%, 22.9%, and 43.3% (Angus); 24.9%, 15.2%, and 79.2% (Hereford); and 26.4%, 32.5%, and 46.2% (Simmental), respectively. Genetic correlations between labs across breeds ranged from 0.79 to 0.95 for IMF, 0.26 to 0.94 for SFD, and 0.78 to 0.98 for LMA. The impact of the observed heterogeneity of variance between labs on genetic evaluation requires further study.

## INTRODUCTION

The use of ultrasound to measure beef carcass traits has been around since the 1950s (Stouffer and Westervelt, 1977). Beef carcass ultrasound can be used to measure longissimus muscle area (LMA), rib fat, intramuscular fat (IMF), and rump fat on live cattle. For U.S. beef cattle genetic evaluations, ultrasound images are collected by an Ultrasound Guidelines Council (UGC) accredited field technician and these images are then interpreted by one of three UGC-certified imaging laboratories. These imaging laboratories are Centralized Ultrasound Processing Lab in Ames, IA; International Livestock Image Analysis in Harrison, AR; and UltraInsights in Pierce, CO. Lab technicians interpret the carcass ultrasound images and send the results to breed associations. Breed associations are then able to utilize the carcass ultrasound data in genetic evaluations (MacNeil and Northcutt, 2008). The use of the ultrasound data has contributed to increased accuracy of selection and, therefore, faster genetic change in carcass traits. For example, from 1998, when the centralized ultrasound processing procedure as described above was implemented, to 2018, the American Angus Association (AAA) has observed an average increase of 0.31 in U.S. Department of Agriculture marbling score and 2.9 cm^2^ in LMA (American Angus Association, 2020).

Currently, the technician and imaging laboratory are included as part of the contemporary group that is used as a fixed effect when estimating breeding values in National Cattle Evaluation (NCE). Because field technicians are certified by the UGC, it is assumed that technician does not affect carcass traits measured by ultrasound and, therefore, the variance due to technician is negligible. However, this assumption has not been rigorously evaluated and very little is known about the contribution of the technician to the variation of ultrasound carcass phenotypes. Similarly, the contribution of imaging lab to the variation of ultrasound carcass phenotypes is also unknown and technicians often report data to the same laboratory. An assumption of the NCE is that additive genetic and residual variances are homogeneous (Van Vleck, 1987), yet this assumption would be false if these variance components were heterogeneous among labs and/or technicians. One consequence of heterogeneous additive genetic variation is that different ranges of estimated breeding values (EBVs) would be observed among laboratories and technicians since technicians often report data to the same laboratory. Selection would favor animals evaluated by technicians and laboratories with more variable EBVs, leading to decreased genetic change if this increased variability is not associated with the additive genetic variance (Hill, 1984; Vinson, 1987).

The hypothesis was that the technician and laboratory contribute to the variation in carcass traits measured by ultrasound. To test this hypothesis, the variance was partitioned into technicians, additive genetics, contemporary groups within technicians, and residual effects for LMA, subcutaneous fat depth (SFD), and percentage of IMF as measured by ultrasound. If the technician did not contribute to variation in carcass traits measured with ultrasound, then technician variance should be negligible. Second, the heterogeneity of residual variance among labs interpreting each of the above three traits was tested. Third, ultrasound carcass traits interpreted by each lab were treated as different traits. This allowed genetic correlations between labs for each trait to be estimated. If the lab was not contributing to variation in ultrasound carcass measurements, then these genetic correlations should not differ from 1.

## MATERIALS AND METHODS

Data were curated from existing information stored by beef breed associations; thus, approval from the South Dakota State University Institutional Animal Care and Use Committee was not required. Ultrasound carcass data from 2015 to 2017 were provided by the AAA (*n* = 281,982), American Hereford Association (AHA; *n* = 49,602), and American Simmental Association (ASA; *n* = 59,576) for a total of 391,160 records. The carcass ultrasound data received by the breed associations were the interpretations made by imaging laboratories that would be used to calculate EBVs for carcass traits. The data used in this evaluation were exactly as provided by the breed association and without any preadjustment. Contemporary group, technician, imaging laboratory, LMA, SFD, and IMF were provided when available on each animal in the data set (Table 1). Subcutaneous fat depth was defined as rib fat by the AHA and ASA and as a combination of rib and rump fat by the AAA. Technician and laboratory identification were coded to maintain anonymity. Technicians certified by the UGC collected all ultrasound images. A total of 93, 121, and 87 technicians collected the ultrasound data used in this study for the AAA, AHA, and ASA, respectively. In this analysis, technicians may be greater than the actual number of individuals due to the assignment of a new technician identification code when a technician changed imaging technologies (Becky Hays, UltraInsights Processing Lab, Inc., personal communication). The median number of ultrasound images collected by each technician was 792, 198, and 104 for data collected for the AAA, AHA, and ASA, respectively. Only data interpreted by the three UGC-certified laboratories (Centralized Ultrasound Processing Lab, International Livestock Image Analysis, and UltraInsights) were analyzed. Contemporary groups were defined by each breed association and included the effects of herd, date of scanning, and sex. Specific management codes were not provided and are assumed to be incorporated into the definition of the contemporary group by the breed association. The age of the animal when scanned was not included in our analysis. Ages of the animal when ultrasound scans are collected to be used for genetic evaluations were 320–460 d for AAA, 270–500 d for ASA, and 301–530 d for AHA. Therefore, data used herein were limited to that collected from animals scanned within these age ranges. A three-generation pedigree for each animal was also provided. Data from each breed association were analyzed separately.

**Table 1.** Description of data used to assess sources of variation in carcass traits measured with ultrasound

Breed	Trait	Interpretation laboratory	Number of scanning technicians—contemporary groups	Number of records	Phenotypic mean	Phenotypic SD	Phenotypic minimum–maximum
Angus	LMA, cm^2^	1	61–2,435	34,946	78.2	15.2	32.5–132.9
		2	14–1,641	14,719	79.1	16.3	29.0–130.0
		3	18–1,415	16,288	75.8	13.8	34.6–123.2
	SFD, mm	1	61–2,435	34,952	6.79	2.77	0.28–23.01
		2	14–1,641	14,719	7.36	2.80	1.40–23.09
		3	18–1,415	16,288	6.73	2.72	0.48–20.22
	IMF, %	1	61–2,435	34,960	4.14	1.30	0.53–12.09
		2	14–1,641	14,719	4.67	1.31	1.04–10.69
		3	18–1,415	16,288	4.72	1.51	1.04–10.32
Hereford	LMA, cm^2^	1	45–2,211	23,122	70.6	14.3	29.0–129.7
		2	12–1,496	11,490	69.3	15.5	28.3–129.7
		3	9–865	8,546	65.8	13.9	27.1–115.5
	SFD, mm	1	45–2,214	21,465	5.74	2.59	0.51–19.05
		2	13–1,499	10,366	5.96	2.51	1.02–19.81
		3	9–865	7,914	5.62	2.71	0.76–22.10
	IMF, %	1	45–2,209	23,120	3.00	0.98	0.32–8.49
		2	12–1,498	11,492	3.45	0.76	0.47–8.53
		3	9–867	8,568	3.46	1.20	1.13–9.98
Simmental	LMA, cm^2^	1	53–1,963	25,799	86.0	14.7	35.5–134.2
		2	11–780	6,018	80.5	16.2	35.0–138.1
		3	23–1,675	16,481	80.3	15.6	27.1–134.8
	SFD, mm	1	53–1,963	25,799	5.72	2.40	1.00–23.00
		2	11–780	6,018	5.47	2.07	1.00–17.00
		3	23–1,675	16,481	5.07	2.34	1.00–20.00
	IMF, %	1	53–1,963	25,799	3.06	1.02	0.48–9.27
		2	11–780	6,018	3.72	0.81	0.53–9.37
		3	23–1,675	16,481	3.57	1.14	1.14–9.27

Variance components were estimated within lab because technicians often reported ultrasound images to the same laboratory, resulting in a lack of independence between technician and lab. Homogeneity of residuals within technicians, after correction for contemporary group effects, was tested using Bartlett’s test. Contemporary groups were nested within technicians because only one technician scanned each contemporary group. Multiple-trait derivative-free restricted maximum likelihood was utilized for the estimation of (co)variance components and genetic correlations (Boldman et al., 1995). Convergence was assumed when the variance of the −2 log L in the simplex was less than 1 × 10^−10^. Convergence to a global maximum was confirmed through multiple analyses, with different starting values, converging to a similar log likelihood. Univariate animal models were fitted for each trait (LMA, SFD, and IMF) and lab combination from each breed for variance component estimation (in total, nine models per breed). For the AHA and ASA data, pedigrees included all sires, dams, grandsires, and granddams. A total of 87,339 animals and 5,008 sires were included in the pedigree for the AHA data. For the ASA data, 79,513 animals and 3,902 sires were included in the pedigree file. Only 157 (mean *F* = 0.17) and 228 (mean *F* = 0.028) animals had nonzero inbreeding coefficients in the AHA and ASA pedigrees, respectively. The analyses did not include inbreeding. Unlike the pedigrees for AHA and ASA, the AAA pedigree was formulated using sires and maternal grandsires. This allowed more Angus animals with records to be included in the analysis because the number of maternal grandsires was less than the number of dams. A total of 78,149 animals and 5,007 sires were included in the AAA pedigree file. None of these animals had nonzero inbreeding coefficients. Variance components were estimated fitting the model:

$$\begin{array}{l} y_{ijk}=\mu +t_{i}+c_{ij}+a_{ijk}+e_{ijk} \end{array}$$

where $y_{ijk}\;$ is the phenotype of the carcass ultrasound trait for the kth animal; μ is the overall mean applied to all observations; $t_{i}$ is a random effect of the ith technician; $c_{ij}$ is a random effect of the jth contemporary group scanned by the ith technician; $a_{ijk}$ is a random effect of additive genetics by the kth animal; and $e_{ijk}$ is a residual deviation from the model effects. Effects were assumed to be normally distributed as follows:

$$\begin{array}{ll} & t∼N(0,I\;\sigma {\;}_{t}^{2})\;\;\;c∼N(0,I\;\sigma {\;}_{c}^{2})\;\;\;a∼N(0,A\;\sigma {\;}_{a}^{2})\;\;\\ & e∼N(0,I\;\sigma {\;}_{e}^{2}) \end{array}$$

where *t* is a random effect of the technician; *c* is a random effect of the contemporary group; *a* is a random effect of the animal; *I* is the identity matrix; *A* is the animal additive numerator relationship matrix$;\sigma_{t}^{2}$ is technician variance; $\sigma_{c}^{2}$ is contemporary group variance; $\sigma_{a}^{2}$ is additive genetic variance; and $\sigma_{e}^{2}$ is residual variance. To estimate the contribution of imaging laboratory to carcass traits measured by ultrasound, genetic correlations were estimated between each pair of the three laboratories for each trait (e.g., SFD for lab 1 and SFD for lab 2). Trivariate analyses were conducted, in which data from each imaging laboratory were considered a different trait to obtain the estimates of these genetic correlations. These analyses used the estimates of the variance components from the univariate analyses as starting points for estimating the covariance components. Thus, the model was:

$$\left[\begin{array}{c} y_{1}\\ y_{2}\\ y_{3} \end{array}\right]=\left[\begin{array}{c} 1\;\mu \;\\ 1\;\mu \;\\ 1\;\mu \; \end{array}\right]+\left[\begin{array}{c} Z_{1}{\text{t}}_{1}\\ Z_{2}{\text{t}}_{2}\\ Z_{3}{\text{t}}_{3} \end{array}\right]+\left[\begin{array}{c} Z_{4}{\text{c}}_{1}\\ Z_{5}{\text{c}}_{2}\\ Z_{6}{\text{c}}_{3} \end{array}\right]+\left[\begin{array}{c} Z_{7}{\text{u}}_{1}\\ Z_{8}{\text{u}}_{2}\\ Z_{9}{\text{u}}_{3} \end{array}\right]+\left[\begin{array}{c} e_{1}\\ e_{2}\\ e_{3} \end{array}\right]$$

where $Z_{i}$ = incidence matrices relating the random effects of the technician ($t_{i}$*, i* = 1–3), contemporary group within technicians ($c_{j}$*, j*= 4–6), and animal ($u_{k}$*, k* = 7–9) to the data and the random residual effects ($e_{l}$). The random effects were assumed to have null means and variances as follows:

$$\begin{array}{l} Var\left[\begin{array}{c} t_{1}\\ t_{2}\\ t_{3} \end{array}\right]=\;\left[\begin{array}{ccc} I\sigma_{t_{1}}^{2} & 0 & 0\\ 0 & I\sigma_{t_{2}}^{2} & 0\\ 0 & 0 & I\sigma_{t_{3}}^{2} \end{array}\right],Var\left[\begin{array}{c} c_{1}\\ c_{2}\\ c_{3} \end{array}\right]=\;\left[\begin{array}{ccc} I\sigma_{c_{1}}^{2} & 0 & 0\\ 0 & I\sigma_{c_{2}}^{2} & 0\\ 0 & 0 & I\sigma_{c_{3}}^{2} \end{array}\right],Var\left[\begin{array}{c} e_{1}\\ e_{2}\\ e_{3} \end{array}\right]=\;\left[\begin{array}{ccc} I\sigma_{e_{1}}^{2} & 0 & 0\\ 0 & I\sigma_{e_{2}}^{2} & 0\\ 0 & 0 & I\sigma_{e_{3}}^{2} \end{array}\right]\text{, and}\\ Var\left[\begin{array}{c} \mu_{1}\\ \mu_{2}\\ \mu_{3} \end{array}\right]=\;\left[\begin{array}{ccc} A\sigma_{a_{1}}^{2} & A\sigma_{\mu_{1}\mu_{2}} & A\sigma_{\mu_{1}\mu_{3}}\\ A\sigma_{\mu_{2}\mu_{1}} & A\sigma_{a_{2}}^{2} & A\sigma_{\mu_{2}\mu_{3}}\\ A\sigma_{\mu_{3}\mu_{1}} & A\sigma_{\mu_{3}\mu_{2}} & A\sigma_{a_{3}}^{2} \end{array}\right] \end{array}$$

Standard errors for the estimates of genetic correlations were calculated as follows (Bijma and Bastiaansen, 2014):

$$\begin{array}{l} SE\left(r_{g}\right)=\sqrt{\frac{\frac{1}{r_{ik}^{2}r_{jk}^{2}}+\left(1+\frac{0.5}{r_{ik}^{4}}+\frac{0.5}{r_{jk}^{4}}-\frac{2}{r_{ik}^{2}}-\frac{2}{r_{jk}^{2}}\right)r_{g}^{2}+r_{g}^{4}}{N-1}} \end{array}$$

where $r_{g}$ is the genetic correlation; $r_{ik}$ is the average accuracy of EBVs for *N* sires for trait *k* interpreted by lab *i*; $r_{jk}$ is the average accuracy of EBVs for sires for trait *k* interpreted by lab *j*; and *N* is the number of sires with images interpreted by both labs *i* and *j*. Spearman’s rank correlations were calculated between sire EBVs with images interpreted by both labs *i* and *j*. Spearman’s correlations assessed the degree of reranking of sire EBVs associated with imaging laboratory. Within contemporary groups, heritability (*h*^2^) estimates of ultrasound carcass traits among labs were calculated as follows.

$$\begin{array}{l} h^{2}=\frac{\sigma_{a}^{2}}{\sigma_{a}^{2}+\sigma_{e}^{2}} \end{array}$$

The technician was not included in the denominator of the above equation because only one technician would collect scans for each contemporary group. The ultrasound technician and contemporary group were not independent of the imaging laboratory; therefore, variance components due to imaging laboratory were unable to be partitioned.

## RESULTS

The total numbers of records for each trait used were 65,950, 43,380, and 48,300 from AAA, AHA, and ASA, respectively (Table 1). The numbers of technicians that provided data to each lab were 61, 14, and 65, respectively. The average contemporary group sizes for AAA, AHA, and ASA were 11.6, 9.4, and 12.8 animals, respectively. Data received from lab 2 (8.2 animals per contemporary group) were generally from smaller contemporary groups than data that were received from labs 1 (12.8 animals per contemporary group) and 3 (9.8 animals per contemporary group).

### Variance Component Analysis

For all traits analyzed in all breeds and labs, the contemporary group consistently explained the greatest fraction of phenotypic variance (Tables 2–4). The technician was generally the second most important contributor to phenotypic variance, explaining 4–27% of the phenotypic variation across all breeds and laboratories. A clear trend was not consistently observed for the percentage of variation explained by the technician across all labs and breeds. However, technician variation tended to explain more of the phenotypic variation than the additive genetic variance explained for LMA across labs and breeds, except for Angus LMA interpreted by lab 3 and Hereford LMA interpreted by labs 2 and 3 (Table 2). Neither technician nor additive genetics consistently explained more of the phenotypic variation for SFD and IMF across all labs and breeds (Tables 3 and 4). Taken together, this indicates that technician variation explained part of the phenotypic variation for LMA, SFD, and IMF across all labs and breeds. Often, technician variation explained as much or more phenotypic variation than additive genetics. These results demonstrate that technician was contributing to LMA, SDF, and IMF variation as measured by ultrasound in three of the largest U.S. beef breeds.

**Table 2.** Genetic, technician, contemporary group, and residual variance components for LMA (cm^2^)

		Variance components and percentages of phenotypic variance							
Breed	Lab	$\sigma_{a}^{2}$	%	$\sigma_{t}^{2}$	%	$\sigma_{c:t}^{2}$	%	$\sigma_{e}^{2}$	%
Angus	Lab 1	16.87	7 ± 1	53.98	23 ± 4	124.13	54 ± 3	35.06	15 ± 1
	Lab 2	16.65	6 ± 1	42.58	16 ± 6	162.95	61 ± 4	45.10	17 ± 1
	Lab 3	17.41	9 ± 1	13.40	7 ± 3	129.10	68 ± 2	29.28	15 ± 1
Hereford	Lab 1	18.85	9 ± 1	34.24	17 ± 4	120.75	59 ± 3	30.50	15 ± 1
	Lab 2	20.45	8 ± 1	15.57	6 ± 3	169.03	70 ± 2	35.97	15 ± 1
	Lab 3	14.75	8 ± 1	8.14	4 ± 3	143.16	74 ± 2	28.11	14 ± 1
Simmental	Lab 1	27.31	13 ± 1	57.21	26 ± 5	93.89	43 ± 3	38.60	18 ± 1
	Lab 2	33.35	13 ± 2	60.64	23 ± 8	126.81	49 ± 5	40.31	15 ± 2
	Lab 3	30.57	12 ± 1	49.98	20 ± 6	133.84	55 ± 4	30.67	13 ± 1

% = percentage of phenotypic variance explained by the variance component ± SE; $\sigma_{a}^{2}$ = additive genetic variance; $\sigma_{t}^{2}$ = technician variance; $\sigma_{c:t}^{2}$ = contemporary group variance; and $\sigma_{e}^{2}$ = residual variance.

**Table 3.** Genetic, technician, contemporary group, and residual variance components for SFD (mm)

		Variance components and percentages of phenotypic variance							
Breed	Lab	$\sigma_{a}^{2}$	%	$\sigma_{t}^{2}$	%	$\sigma_{c:t}^{2}$	%	$\sigma_{e}^{2}$	%
Angus	Lab 1	0.98	13 ± 1	1.48	19 ± 3	3.58	47 ± 2	1.64	21 ± 1
	Lab 2	0.87	11± 1	0.92	12 ± 5	4.26	54 ± 3	1.79	23 ± 2
	Lab 3	1.08	15 ± 2	1.44	19 ± 6	3.46	47 ± 4	1.42	19 ± 2
Hereford	Lab 1	0.86	13 ± 1	0.64	10 ± 2	3.18	47 ± 2	2.04	30 ± 1
	Lab 2	0.80	13 ± 2	0.33	5 ± 3	3.27	52 ± 2	1.93	31 ± 2
	Lab 3	0.74	10 ± 2	1.68	23 ± 9	3.16	43 ± 5	1.75	24 ± 3
Simmental	Lab 1	1.43	25 ± 2	1.15	20 ± 4	1.58	28 ± 2	1.59	28 ± 2
	Lab 2	0.92	22 ± 3	0.70	16 ± 6	1.35	31 ± 3	1.32	31 ± 3
	Lab 3	0.93	17 ± 2	1.24	23 ± 6	2.17	39 ± 3	1.15	21 ± 2

% = percentage of phenotypic variance explained by the variance component ± SE; $\sigma_{a}^{2}$ = additive genetic variance; $\sigma_{t}^{2}$ = technician variance; $\sigma_{c:t}^{2}$ = contemporary group variance; and $\sigma_{e}^{2}$ = residual variance.

**Table 4.** Genetic, technician, contemporary group, and residual variance components for percentage of IMF (%)

		Variance components and percentages of phenotypic variance							
Breed	Lab	$\sigma_{a}^{2}$	%	$\sigma_{t}^{2}$	%	$\sigma_{c:t}^{2}$	%	$\sigma_{e}^{2}$	%
Angus	Lab 1	0.34	20 ± 2	0.43	25 ± 4	0.56	33 ± 2	0.37	22 ± 1
	Lab 2	0.52	30 ± 3	0.21	12 ± 5	0.73	43 ± 3	0.26	15 ± 2
	Lab 3	0.51	22 ± 2	0.33	15 ± 5	1.03	45 ± 3	0.41	18 ± 2
Hereford	Lab 1	0.16	16 ± 1	0.21	22 ± 4	0.37	34 ± 2	0.27	28 ± 2
	Lab 2	0.15	26 ± 2	0.07	12 ± 5	0.23	39 ± 3	0.13	23 ± 2
	Lab 3	0.24	17 ± 2	0.20	14 ± 6	0.69	48 ± 4	0.32	22 ± 2
Simmental	Lab 1	0.28	27 ± 2	0.27	27 ± 4	0.26	25 ± 2	0.23	22 ± 2
	Lab 2	0.17	26 ± 3	0.10	16 ± 6	0.22	34 ± 3	0.16	25 ± 3
	Lab 3	0.31	24 ± 2	0.18	14 ± 4	0.55	42 ± 2	0.26	20 ± 2

% = percentage of phenotypic variance explained by the variance component ± SE; $\sigma_{a}^{2}$ = additive genetic variance; $\sigma_{t}^{2}$ = technician variance; $\sigma_{c:t}^{2}$ = contemporary group variance; and $\sigma_{e}^{2}$ = residual variance.

### Consistency of Technician Variance Across Labs and Breeds

Differences in the percentage of variation explained by the technician were observed among imaging laboratories. The technician explained the least amount of phenotypic variation for SFD for lab 2 across all breeds (Table 3) and IMF for lab 2 for data submitted to the AAA and AHA (Table 4). Technicians reporting data to lab 1 explained the highest amount of variation for LMA and IMF relative to the other laboratories (Tables 2 and 4). Technicians reporting to lab 3 explained the least amount of technician variation across all breeds for LMA. The amount of variation explained by the technician differed between breeds. Technician contributed on average the least amount of variation for data submitted to the AHA relative to the other two breeds except for SFD reported by lab 3. It is noteworthy that the range among interpretation laboratories in variance attributable to the technician is less within breeds than between breeds.

### Homogeneity of Residual Variation Among Imaging Labs

Within breeds, estimates of additive genetic variance for LMA, SFD, and IMF differed (range divided by mean) among laboratories for all breeds by 4.5%, 21.5%, and 39.4% (Angus); 31.6 %, 15.0%, and 49.1% (Hereford); and 19.9%, 46.6%, and 55.3% (Simmental), respectively. Likewise, the estimates of residual variance for LMA, SFD, and IMF differed among laboratories by 43.4%, 22.9%, and 43.3 % (Angus); 24.9%, 15.2%, and 79.2% (Hereford); and 26.4%, 32.5%, and 46.2% (Simmental), respectively. The homogeneity of residuals across technicians was tested for each trait and breed combination (*n* = 9). Tests of homogeneity of variance consistently demonstrated that residuals were not homogeneous across technicians for all traits and breeds (Bartlett’s Test *P* < 0.0001). This test of homogeneity confirmed heterogeneous residual variance among imaging labs, which was consistent with the estimates of residual variation among labs across all breeds and traits (Tables 2–4). Residual variation was not homogeneous among the three imaging laboratories interpreting LMA, SDF, and IMF ultrasound scans for the AAA, AHA, and ASA breeds.

### Heritability Estimates

In order to compare results with previous literature, heritabilities were estimated using the variance components for animal and residual effects in the denominator (Table 5). These estimates were, as expected, greater than the percentages of variation attributed to animal effects in Tables 2–4. Heritability estimates for ultrasound carcass traits across breeds were moderate to high, ranging from 0.25 to 0.67. Within contemporary groups, heritability estimates for ultrasound LMA ranged from 0.27 to 0.50, SFD estimates ranged from 0.25 to 0.47, and IMF estimates ranged from 0.34 to 0.67 across breeds. Intramuscular fat had the highest heritability estimates among carcass traits for each breed. These results agree with previous literature of reported heritability estimates of 585 ultrasound carcass measurements (Robinson et al., 1993; Reverter et al., 2000; Kemp et al., 2002).

**Table 5.** Estimates of heritability for carcass traits measured on ultrasonic images when the phenotypic variance is assumed to equal $\;\sigma_{a}^{2}+\sigma_{e}^{2}$

		Ultrasound carcass trait		
Breed	Lab	LMA	SFD	IMF
Angus	1	0.32 ± 0.02	0.37 ± 0.02	0.48 ± 0.02
	2	0.27 ± 0.03	0.33 ± 0.03	0.67 ± 0.04
	3	0.38 ± 0.03	0.43 ± 0.03	0.55 ± 0.04
Hereford	1	0.35 ± 0.02	0.26 ± 0.02	0.34 ± 0.02
	2	0.35 ± 0.03	0.25 ± 0.03	0.49 ± 0.03
	3	0.34 ± 0.03	0.29 ± 0.03	0.42 ± 0.03
Simmental	1	0.41 ± 0.02	0.47 ± 0.02	0.55 ± 0.02
	2	0.45 ± 0.05	0.41 ± 0.05	0.52 ± 0.05
	3	0.50 ± 0.03	0.45 ± 0.03	0.54 ± 0.03

### Genetic Correlations Between Laboratories

Genetic correlations between imaging laboratories for each trait within breeds were generally high (Tables 6–8). Genetic correlations between all pairs of labs within breeds ranged from 0.26 to 0.98 across all ultrasound measurements. Hereford SFD genetic correlations were the lowest estimates compared to all other breeds and carcass traits ranging from 0.26 to 0.70 (Table 8). Genetic correlation estimates for all other traits and breeds were ≥0.78. Genetic correlations were highest between labs 1 and 3 for all traits and breeds except for IMF and SFD in Angus and Hereford, where correlations were highest between labs 1 and 2 (Tables 6–8). Spearman’s rank correlations between EBVs based on image interpretations from different labs were also generally high and positive (Tables 6–8). Rank correlations were above 0.90 with the exception of LMA between labs 1 and 2 for Simmental data and all lab combinations for Hereford SFD. Spearman rank correlations indicate little change in sire rankings based on EBVs when different labs interpret ultrasound images, with the exception of SFD reported to the AHA. Taken together, imaging lab genetic correlations were high but often not equal to 1, suggesting that the lab had less impact on EBV estimation or ranking of genetic merit than the technician. However, lower correlations were observed between labs for some trait–breed combinations, in particular, Hereford SFD observations.

**Table 6.** Genetic correlations (±SE) between labs interpreting ultrasound LMA (below diagonals) and Spearman rank correlations of EBVs for LMA of sires with progeny interpreted by each pair of laboratories (above diagonals) estimated within Angus, Hereford, and Simmental breeds

Breed	Lab	Lab 1	Lab 2	Lab 3
Angus	Lab 1		0.99 (417)	0.99 (501)
	Lab 2	0.94 ± 0.04		0.99 (327)
	Lab 3	0.96 ± 0.04	0.94 ± 0.04	
Herford	Lab 1		0.95 (245)	1.00 (199)
	Lab 2	0.92 ± 0.06		0.96 (251)
	Lab 3	0.98 ± 0.06	0.88 ± 0.06	
Simmental	Lab 1		0.88 (341)	0.94 (510)
	Lab 2	0.78 ± 0.06*		0.93 (320)
	Lab 3	0.85 ± 0.05	0.80 ± 0.06*	

Number of sires is shown in parentheses.

*z-test *of estimate against 1.0 is rejected at P* = 0.05.

**Table 7.** Genetic correlations (±SE) between labs interpreting ultrasound subcutaneous fat (below diagonals) and Spearman’s rank correlations of EBVs for SFD of sires with progeny interpreted by each pair of laboratories (above diagonals) estimated within Angus, Hereford, and Simmental breeds

Breed	Lab	Lab 1	Lab 2	Lab 3
Angus	Lab 1		0.99 (418)	0.98 (501)
	Lab 2	0.93 ± 0.04		0.98 (327)
	Lab 3	0.92 ± 0.04*	0.92 ± 0.04*	
Hereford	Lab 1		0.82 (232)	0.77 (185)
	Lab 2	0.70 ± 0.11*		0.49 (238)
	Lab 3	0.58 ± 0.14*	0.26 ± 0.14*	
Simmental	Lab 1		0.95 (341)	0.99 (510)
	Lab 2	0.82 ± 0.05*		0.93 (341)
	Lab 3	0.94 ± 0.04	0.79 ± 0.06*	

Number of sires is shown in parentheses.

*z-test *of estimate against 1.0 is rejected at P* = 0.05.

**Table 8.** Genetic correlations (±SE) between labs interpreting ultrasound IMF (below diagonals) and Spearman rank correlations of EBVs for percentage of IMF of sires with progeny interpreted by each pair of laboratories (above diagonals) estimated within Angus, Hereford, and Simmental breeds

Breed	Lab	Lab 1	Lab 2	Lab 3
Angus	Lab 1		0.99 (418)	0.99 (501)
	Lab 2	0.95 ± 0.03		0.97 (327)
	Lab 3	0.94 ± 0.03*	0.89 ± 0.03*	
Hereford	Lab 1		0.97 (245)	0.97 (200)
	Lab 2	0.89 ± 0.06*		0.93 (251)
	Lab 3	0.87 ± 0.07*	0.80 ± 0.06*	
Simmental	Lab 1		0.94 (341)	0.97 (320)
	Lab 2	0.79 ± 0.05*		0.96 (510)
	Lab 3	0.88 ± 0.04*	0.87 ± 0.05*	

Number of sires is shown in parentheses.

*z-test *of estimate against 1.0 is rejected at P* = 0.05.

## DISCUSSION

### Technician Variance

Each lab utilizes a different technology to interpret ultrasound images, which may be contributing to the lower proportion of phenotypic variance explained by the technician for IMF and SFD interpreted by lab 2. Although imaging software differs among laboratories, this study could not separate the effects of imaging software from other laboratory effects due to the structure of the data. The specific reasons for this interlab variation are, therefore, unknown. The technician explained a higher percentage of the variation for LMA than SFD for labs 1 and 2. Ultrasound estimates for SFD are often more accurate than LMA because of the difficulty in measuring the area of the longissimus muscle (Perkins et al., 1992). Generally, SFD is easier to measure than LMA. As shown by Greiner et al. (2003), the definition of the outline of the longissimus muscle that makes up the LMA can be affected by the amount of fat present. The more the backfat, the less easily the ventral edge of the longissimus muscle can be defined, thus leading to less-accurate interpretations. Also, the LMA has the shape of an elongated rectangle or oval with an area that can range from 70 to 116 cm^2^, whereas SFD is a linear measurement that ranges from 10 to 30 mm. Considering these factors may help interpret the lower overall proportion of phenotypic variance explained by the technician for SFD versus LMA. Interestingly, this relationship did not hold for lab 3, where the technician explained a greater percentage of variation for SFD than LMA. It is unclear whether this result occurred because of differences in technology, laboratory technician, or other factors.

The proportion of phenotypic variance explained by the technician was not zero for all trait, lab, and breed combinations. In some cases, the technician even explained a larger proportion of variance than additive genetics. These results suggest that improved technician training, ultrasound equipment, or both would improve genetic evaluations for LMA, SDF, and IMF as measured by ultrasound. Because the UGC goes to considerable effort in the certification of technicians in order to ensure their consistent performance, this result may be unexpected. Because an individual technician may use more than one particular technology to capture images, some of the variations that have been attributed to technicians may well reflect the variation in technologies rather than in the skill of any particular technician. Within technicians, contemporary groups were generally the single most important source of variation across all interpretation labs, traits, and breeds. This result might be expected because contemporary groups differ due to a number of identifiable factors that would influence carcass ultrasound performance. These identifiable factors would include on-farm management, nutrition, and sex, as well as other less readily identified idiosyncrasies specific to a contemporary group (Park et al., 2018).

### Effect of Imaging Laboratory

Most (21 of 27) genetic correlations between imaging laboratories were ≥0.80, suggesting a lesser contribution of imaging laboratory to variation in the EBVs. Still, 6 of the 27 genetic correlations between laboratories were ≤0.80, with Hereford SFD between labs 2 and 3 having the lowest genetic correlation (0.26). This low correlation may be partly attributed to the different technology utilized by lab 2. Generally, the technician explained a lower percentage of phenotypic variation for SFD and IMF interpreted by lab 2. Furthermore, genetic correlations between labs were generally highest between labs 1 and 3. Although the genetic correlations were high, in many instances, these correlations were different from 1. Taken together, some evidence exists that the imaging laboratory contributes to ultrasound carcass variation, although the contribution of the lab was less than the contribution of the variance of the technician. Robertson (1959) suggested that genetic correlations greater than 0.80 should be treated as the same trait. If this recommendation is followed, only genetic correlations between laboratories for SFD reported to AHA are clearly problematic. Among the six genetic correlations <0.80, SFD for the Hereford data had the lowest correlations. A more stringent criterion of 1 for genetic correlations would lead to the opposite conclusion: imaging laboratory contributed to EBV variation across all traits and breeds. Thus, some evidence has been presented that imaging laboratory may be contributing to heterogeneous additive genetic variation, violating an important assumption of the NCEs.

The cause of Hereford genetic correlations among imaging laboratories being substantially less than anticipated and than those observed for the other breeds cannot be definitively determined from these data. The technician contributed more variance for SFD from lab 3 than the other two labs within data submitted to the AHA, which is in contrast to results for IMF and LMA, where technicians reporting to lab 3 did not contribute more variation than the other two labs. Furthermore, heritability estimates for SFD were consistently lower in Herefords than the other breeds. Based on the estimates of heritability, the imaging laboratories would appear to be internally consistent in their measurement technique for SFD. However, Hereford hides have long been known to be heavier than the hides from other breeds (Butler et al., 1962), and the added thickness may contribute to differences in measurement techniques among the imaging laboratories. Alternatively, these results may be an anomaly. The likelihood response surface relative to these estimates of genetic correlation was flatter than for Angus and Simmental, suggesting that the convergence of restricted maximum likelihood iterations was less sensitive to the particular estimates of the genetic correlations. The degree to which these factors can explain the lower genetic correlations for SFD in Herefords is unknown.

### Heritability Estimates

Heritability estimates were moderate to high, which is expected for carcass traits. Previous literature describing carcass ultrasound traits of *Bos taurus* cattle breeds found heritability estimates ranging from 0.29 to 0.42 for LMA, 0.18 to 0.51 for SFD, and 0.20 to 0.33 for IMF (Reverter et al., 2000; Kemp et al., 2002). These values are comparable to the estimates found in this analysis. The IMF heritability estimates tended to be higher than what previous literature has found, which may be due to the improvement of ultrasound technology or processes for scanning and interpreting IMF. In the present analyses where the technician and the contemporary group were considered as additional random effects (Tables 2–4), the additive genetic variance was a smaller fraction of phenotypic variance (V(p)) because $V\left(\;p\right)=\sigma_{a}^{2}+\sigma_{t}^{2}+\sigma_{c}^{2}+\sigma_{e}^{2}$ and $\sigma_{t}^{2}$ and $\sigma_{c}^{2}\;$were greater than 0. Estimates of additive genetic variance and heritability were often higher in the ASA data than the other two breed data sets. It should be noted that the data set received from ASA likely contained more admixture than data from either AAA or AHA. A difference in the level of admixture could inflate the estimates of additive genetic variance from ASA relative to those from AAA and AHA. It is also possible that the higher level of admixture (crossbreeding) in ASA data inflated technician variance given that herds often use the same technicians each year. This analysis did not include the age of the animal when scanned by the technician. Age is normally included as a covariate in ultrasound carcass genetic evaluations. The likely effects of including age in these analyses would be to reduce the estimates between contemporary groups within technician and residual variances with lesser, if any, effects on the additive genetic and technician estimates of variance. 

### Industry Implications

While these results found variation among ultrasound technicians and laboratories, the underlying causes of this variability cannot be identified. Technician and laboratory variance can stem from a number of factors. Ultrasound machines, transducers, and scanning techniques have been shown to contribute to technician variance (Perkins et al., 1992; Herring et al., 1994; Greiner et al., 2003). Ultrasound equipment used by each technician was not available and, therefore, differences in variation among equipment types were not able to be examined. Understanding the effect of ultrasound equipment on ultrasound carcass phenotypes would be helpful for developing best practices for ultrasound carcass evaluation.

Ultrasound technician and laboratory are potential sources of variation when estimating carcass merit by ultrasound. This study demonstrates that the technician is an important source of variation in carcass phenotypes measured by ultrasound. Technician variance often equaled or exceeded additive genetic variation, showing that improvements in the technician certification process may be needed. Variance among ultrasound technicians should be addressed by future work into the causes of variation among technicians. Reducing this variance will help improve the accuracy of ultrasound carcass measurements and genetic predictions. Genetic correlations between laboratories were generally high, which suggests that they play a lesser role in the contribution of variance to ultrasound measurements than the technician. However, these correlations, especially for SFD in Herefords, were often statistically different from 1, which is the assumption in the beef cattle genetic evaluations. Residual variation was also not homogeneous among laboratories. Further investigation into the consistency of lab performance when interpreting ultrasound carcass images is warranted. The results of this research indicate potential areas for improvement of U.S. beef cattle evaluations using carcass ultrasound measurements, specifically in the certification process for ultrasound technicians and image processing laboratories. Breed associations can use these results to guide the collection of carcass data using ultrasound, resulting in improved genetic evaluations for carcass traits. Until the technician and imaging lab variances can be made more homogeneous, breed associations may consider fitting a heterogeneous variance model to account for differences among technicians and laboratories. Imaging lab contributed to a lesser degree to breeding value estimation and, with the exception of SFD in Hereford cattle, little change in sire rankings based on EBVs was observed when different labs interpreted the ultrasound images for the progeny of the same sires.
